# Scaling COVID-19 against inequalities: should the policy response consistently match the mortality challenge?

**DOI:** 10.1136/jech-2020-214373

**Published:** 2020-11-02

**Authors:** Gerry McCartney, Alastair Leyland, David Walsh, Dundas Ruth

**Affiliations:** 1 Place and Wellbeing Directorate, Public Health Scotland, Glasgow, UK; 2 MRC/CSO Social and Public Health Sciences Unit, University of Glasgow, Glasgow, UK; 3 Glasgow Centre for Population Health, Glasgow, UK

**Keywords:** Epidemiological methods, Health inequalities, Mortality

## Abstract

**Background:**

The mortality impact of COVID-19 has thus far been described in terms of crude death counts. We aimed to calibrate the scale of the modelled mortality impact of COVID-19 using age-standardised mortality rates and life expectancy contribution against other, socially determined, causes of death in order to inform governments and the public.

**Methods:**

We compared mortality attributable to suicide, drug poisoning and socioeconomic inequality with estimates of mortality from an infectious disease model of COVID-19. We calculated age-standardised mortality rates and life expectancy contributions for the UK and its constituent nations.

**Results:**

Mortality from a fully unmitigated COVID-19 pandemic is estimated to be responsible for a negative life expectancy contribution of −5.96 years for the UK. This is reduced to −0.33 years in the fully mitigated scenario. The equivalent annual life expectancy contributions of suicide, drug poisoning and socioeconomic inequality-related deaths are −0.25, −0.20 and −3.51 years, respectively. The negative impact of fully unmitigated COVID-19 on life expectancy is therefore equivalent to 24 years of suicide deaths, 30 years of drug poisoning deaths and 1.7 years of inequality-related deaths for the UK.

**Conclusion:**

Fully mitigating COVID-19 is estimated to prevent a loss of 5.63 years of life expectancy for the UK. Over 10 years, there is a greater negative life expectancy contribution from inequality than around six unmitigated COVID-19 pandemics. To achieve long-term population health improvements it is therefore important to take this opportunity to introduce post-pandemic economic policies to ‘build back better’.

## BACKGROUND

The COVID-19 pandemic has been tracked by daily counting of cumulative numbers of confirmed cases and deaths.^1^ The exponential growth in these numbers across countries has understandably created anxiety and action from public health agencies and governments internationally. Despite initial surveillance and reporting of COVID-19 following standard infectious disease epidemiologic methods, the subsequent reporting of COVID-19 mortality has largely focused on crude death counts, arguably not meeting the ‘rigorous standardisation and quality control of investigative methods [that] are essential in epidemiology’.^2^ A number of particular limitations in the data have prevented a sufficient understanding of the true impact of the pandemic on mortality.

First, the counting of cases of COVID-19 within and between countries has been dependent on the case definition and the changes in that over time. At the beginning of the outbreak in Wuhan, China, cases were defined clinically before virological testing was available. Then, as testing became partially available, cases were defined as people with a travel history from China, or contact with a known case, and a positive virology test. Then, cases were defined as people with a positive virology test irrespective of symptoms or history, although the availability of tests remained restricted.^3^ This is problematic for epidemiological surveillance, because limited availability of testing, and the self-limiting nature of the infection for many, meant that the case count underestimated the true incidence within the population. For COVID-19 deaths, the count was initially based on people who died within hospital who had a positive virological test. This is subsequently being extended in most countries to include coding of deaths based on clinical opinion in all settings. This raises a further issue because many deaths will occur in people who die with, rather than die of, COVID-19.

Deaths occur every day, and simply reporting the *cumulative* number of deaths for any particular cause, be that COVID-19 or anything else, will always reveal a rising trend. Other causes of death are not reported in this way. It is therefore difficult for the public and policymakers to understand how to interpret and compare these to other causes of death.

The COVID-19 deaths reported are crude death counts. They therefore do not take into account the size of the population at risk (as a crude rate does), nor the age and sex structure of the population, in particular, how old the population is (as an age-sex-standardised rate does). In contrast, other causes of death such as cancers and heart disease, and deaths attributable to deprivation, poverty and other political and socioeconomic causes such as austerity are usually measured as differences in such standardised rates, in Years of Life Lost (YLL), or life expectancy contributions.^4^ Finally, the reported crude death counts also do not account for competing causes and how likely people dying from COVID-19 were to have died relatively soon from other causes.^5^ It is therefore difficult to assess the scale of the mortality risk of COVID-19 relative to the background mortality risk in the population.

These problems of interpretation are very important. If COVID-19 generates a substantially higher mortality rate across all or part of the population than would otherwise have been expected, this would support much more radical action to control the pandemic. On the other hand, if COVID-19 has little additional impact on the mortality rate compared to what would otherwise have been expected, then it may be that the negative health impacts of the control measures (eg, due to the impacts of closing down large sectors of the economy^6^) outweigh the positive impact of mitigating the pandemic.

Given the importance of all the above, the aim of this paper is to apply rigorous epidemiological methods using consistent definitions to compare age-sex-standardised mortality and life expectancy contributions of mortality from COVID-19 with the total attributable life expectancy impact of socioeconomic inequality, and with two examples of causes of death which are experienced particularly unequally and which have been the focus of some recent policy attention: suicide and drug-related poisonings.

## METHODS

### Estimating COVID-19 mortality

The UK Government has based decisions on pandemic control measures on the modelling of the Imperial College team on the likely scale and timing of the pandemic. The estimate (published on 16 March 2020) of the number of deaths in an unmitigated epidemic in Great Britain (GB) is between 410 000 and 550 000 (with a best estimate of 510 000), and between 5600 and 48 000 in a fully mitigated epidemic (with a best estimate of 20 000).^7^ Estimated deaths by age group are not provided, but ‘infection fatality ratios’ (the percentage of people who are infected who are then expected to die) for 10-year age bands up to age 79 years, with an open upper age band, are provided. Using the 2018 midyear population data from the Office for National Statistics (ONS) for these age bands for GB and assuming that the proportion of the population in each age group that become infected is uniform, we weighted the deaths in each age group to achieve the total number of deaths estimated by Ferguson *et al.* We fitted a linear regression model to the logarithm of the infection fatality rates in order to estimate the rate for each 5-year, rather than 10-year, age bands up to age 90 years (online supplemental figure S1). These rates were then applied to population data and the total scaled to the estimated deaths in GB under the two scenarios (mitigated and unmitigated). As a sensitivity analysis, we applied the age distribution of the deaths certified with COVID-19 as the underlying cause in England and Wales between March and June 2020^8^ to calculate the life expectancy impact.

10.1136/jech-2020-214373.supp1 Supplementary data

### Estimating suicide, drug poisoning and inequality-related mortality

We obtained population counts for England, Northern Ireland, Scotland and Wales for each year between 2013 and 2017, by 5-year age band and sex, from the Office for National Statistics (ONS), National Records of Scotland (NRS) and the Northern Ireland Statistics and Research Agency (NISRA). We also obtained the count of all deaths by each nation’s deprivation decile by age and sex, and for two specific causes of death (suicide, including events of undetermined intent; and drug poisoning) by age and sex for each year between 2013 and 2017. The ICD10 codes for suicide were X60-X84, Y10-Y34, Y87.0 and Y87.2; and for drug-related poisonings were F11-F16, F18, F19, X40-X44, X60-X64, X85 and Y10-Y14. The drug poisoning deaths are coded using the broader definition, and the suicide coding uses the older (pre-2011) codes. The definitions of suicide and drug poisoning deaths overlap and cannot therefore be summed.^9^ Following Lewer,^10^ we defined deaths due to inequality as all deaths higher than the rate of mortality in the least deprived tenth of the population in each population. This is akin to calculating the population attributable fraction of inequality. Deprivation was measured using IMD2015 for England, WIMD2014 for Wales, SIMD2016 for Scotland and the 2010 Northern Ireland Multiple Deprivation Measure (NIMDM) for Northern Ireland. We also performed a sensitivity analysis which attributed all deaths above the age-specific rate across every population compared to the least deprived tenth of England (as the nation with the lowest mortality rate in the least deprived tenth).

### Estimating the contribution of different causes to crude and standardised mortality rates, and life expectancy

For crude deaths, we simply summed the reported number of deaths in each age band (taking the 5-year mean between 2013 and 2017 for suicide, drug-related and inequality-related deaths). For age-standardised deaths, we applied the age-specific deaths to the 2013 European Standard Population to calculate directly standardised estimates. Overall life expectancy was estimated using the method detailed by Auger *et al* ^11^ based on counts of death and populations in 5-year age groups (together with deaths as age 0, 1–4 and aged 90 and over). For COVD-19 and inequality deaths in Northern Ireland, we had to estimate the distribution between the 0–1 and 1–4 year age bands as this breakdown was not available. The effect of COVID was estimated by adding the predicted deaths under the two scenarios, recalculating the life expectancy and taking the difference. The effect of the other causes on life expectancy was estimated by subtracting the deaths attributable to those causes, recalculating life expectancy and taking the difference.

## RESULTS

Table 1 provides the estimates of the unmitigated and fully mitigated mortality impact of the COVID-19 pandemic by age group for the UK and its nations, based on the Imperial College modelling. It estimates that the majority of deaths will be in the older age groups because the smaller population size is outweighed by the higher mortality rates for these ages. For the UK overall there are estimated to be 195 420 and 7664 deaths among those aged 80+ years in the unmitigated and mitigated scenarios, respectively, with 28% of all deaths occurring under the age of 70 years and 38% of all deaths occurring for those aged 80+ years. The proportions are very similar across the nations.

**Table 1:** Estimates of the potential mortality impact of COVID-19 on different age groups by applying assumptions to the imperial model

Age group (years)	UK age distribution*	Infection Fatality Ratio	UK	GB	England & Wales	Scotland	Northern Ireland
**Estimated number of deaths in each age group if there were a total of 510 000 deaths in GB**							
0 to 4	6%	0.002%	44	42	39	3	1
5 to 9	6%	0.003%	78	76	70	6	2
10 to 14	6%	0.006%	124	120	111	9	4
15 to 19	6%	0.010%	200	194	178	16	6
20 to 24	6%	0.017%	387	376	344	32	11
25 to 29	7%	0.028%	711	691	631	60	19
30 to 34	7%	0.048%	1189	1155	1059	96	34
35 to 39	7%	0.082%	1975	1920	1762	157	56
40 to 44	6%	0.139%	3061	2972	2728	243	89
45 to 49	7%	0.236%	5860	5694	5208	487	166
50 to 54	7%	0.400%	10 310	10 018	9125	893	292
55 to 59	6%	0.678%	16 066	15 611	14 165	1447	455
60 to 64	6%	1.150%	23 316	22 663	20 528	2135	652
65 to 69	5%	1.952%	36 574	35 613	32 378	3235	961
70 to 74	5%	3.311%	59 400	57 930	52 980	4950	1470
75 to 79	3%	5.617%	69 281	67 442	61 520	5922	1839
80 to 84	3%	9.528%	87 980	85 803	78 429	7374	2177
85 to 89	2%	16.164%	91 370	89 177	81 913	7264	2192
90+	1%	35.716%	115 091	112 502	104 240	8262	2589
Total			523 016	510 000	467 409	42 591	13 016
**Estimated number of deaths in each age group if there were a total of 20 000 deaths in GB**							
0 to 4	6%	0.002%	2	2	2	0	0
5 to 9	6%	0.003%	3	3	3	0	0
10 to 14	6%	0.006%	5	5	4	0	0
15 to 19	6%	0.010%	8	8	7	1	0
20 to 24	6%	0.017%	15	15	13	1	0
25 to 29	7%	0.028%	28	27	25	2	1
30 to 34	7%	0.048%	47	45	42	4	1
35 to 39	7%	0.082%	77	75	69	6	2
40 to 44	6%	0.139%	120	117	107	10	3
45 to 49	7%	0.236%	230	223	204	19	7
50 to 54	7%	0.400%	404	393	358	35	11
55 to 59	6%	0.678%	630	612	555	57	18
60 to 64	6%	1.150%	914	889	805	84	26
65 to 69	5%	1.952%	1434	1397	1270	127	38
70 to 74	5%	3.311%	2329	2272	2078	194	58
75 to 79	3%	5.617%	2717	2645	2413	232	72
80 to 84	3%	9.528%	3450	3365	3076	289	85
85 to 89	2%	16.164%	3583	3497	3212	285	86
90+	1%	35.716%	4513	4412	4088	324	102
Total			20 510	20 000	18 330	1670	510

*ONS 2018 mid-population estimate. The population profiles for each nation/GB/UK were used to weight the estimates, but these are not shown because they are very similar to the estimates for the UK overall. GB, Great Britain; UK, United Kingdom.

Table 2 shows the annual (5-year mean) age-specific mortality counts for suicide, drug poisoning and inequality-related mortality by nation. These causes have a much younger age distribution of deaths compared to COVID-19, with the highest UK age-specific mortality counts being among 45–49 year olds for suicides and 40–44 year olds for drug poisonings. The peak age for crude mortality due to inequality was 80–84 years but there was a broad age distribution. Suicide and drug poisonings have lower overall counts compared to mitigated COVID-19. However, deaths due to inequality are seven times those of mitigated COVID-19. The crude mortality is substantially higher for men than women for suicide, drug poisonings and inequality (online supplemental tables S1 and S2). The modelling of COVID-19 deaths was not available separately by sex. The sensitivity analysis which used the least deprived tenth of the population of England as the comparator increased the number of deaths attributable to inequality slightly as the least deprived tenth of England has lower mortality than the equivalents across the other nations. However, the impact on the overall results is small (online supplemental table S3). Suicide, drug poisonings and inequalities all generate mortality every year, and cumulatively over a few years amount to greater total crude deaths than COVID-19. For example, in 3.5 years the total death count for inequality-related deaths overtakes the number of COVID-19 deaths in the UK in the completely unmitigated scenario.

**Table 2:** Estimated number of deaths due to suicide, drug poisonings and inequality-related deaths by age (2013–2017 annualised mean, total population)

	Drug poisonings					Suicide					Inequality				
Age group (years)	UK	GB	E&W	Sco	NI	UK	GB	E&W	Sco	NI	UK	GB	E&W	Sco	NI
0–4	1	1	1	0	0	4	4	3	1	0	1040	1043	983	60	−3
5–9	1	1	1	0	0	1	1	1	0	0	97	93	92	1	4
10–14	3	3	2	1	0	20	18	15	3	2	58	54	45	9	4
15–19	63	59	46	13	4	195	182	161	20	14	280	255	222	33	25
20–24	194	181	150	31	13	395	364	321	43	31	266	221	152	69	46
25–29	356	339	271	67	17	461	428	370	58	33	583	562	440	122	21
30–34	528	508	395	114	19	519	483	422	61	36	1160	1122	890	232	38
35–39	672	655	516	139	17	531	500	432	68	31	1958	1880	1557	324	78
40–44	734	719	558	161	15	663	633	547	86	31	3189	3119	2651	467	70
45–49	632	618	490	127	14	737	703	611	92	34	5032	4853	4226	627	179
50–54	467	456	365	91	11	684	653	574	80	31	6470	6287	5412	876	183
55–59	284	277	232	46	6	523	504	442	62	19	8377	8149	6993	1156	228
60–64	189	184	158	26	5	365	350	309	41	15	11 473	11 197	9707	1490	276
65–69	116	115	100	15	2	287	278	249	29	9	16 053	15 629	13 609	2019	424
70–74	74	72	63	9	2	209	204	180	23	6	18 497	18 070	15 649	2421	426
75–79	56	55	49	6	1	162	158	142	16	4	21 398	20 961	18 371	2591	436
80–84	41	40	39	2	1	128	127	118	9	1	22 305	21 982	19 501	2481	322
85–89	29	29	27	2	0	95	94	88	5	1	17 154	16 886	15 194	1692	268
90+	22	22	21	1	0	56	56	52	4	0	11 957	11 799	11 319	481	158
Total	4460	4334	3483	850	126	6038	5739	5039	701	298	147 346	144 164	127 013	17 150	3182

E&W, England & Wales; GB, Great Britain; NI, Northern Ireland; Sco, Scotland; UK, United Kingdom.

Table 3 compares the crude mortality, age-standardised mortality and life expectancy contributions of the different causes. The negative contribution to life expectancy for drug, suicide and inequality-related deaths is much greater than the contribution from the fully mitigated COVID-19 mortality. The contribution to life expectancy of inequality-related deaths is higher, and the contribution of COVID-19 deaths lower, in Scotland and Northern Ireland compared to the UK overall. The sensitivity analysis using the age distribution of the underlying COVID-19 deaths in England and Wales (online supplemental table S4) shows very similar results to those based on the Imperial College modelling, stemming from the predicted and actual age distributions being very similar.

**Table 3:** Comparison of the impacts of COVID and other causes on mortality indices

Age group (years)	UK	GB	England & Wales	Scotland	Northern Ireland
**Crude deaths**					
COVID-19 unmitigated	523 016	510 000	467 409	42 591	13 016
COVID-19 fully mitigated	20 510	20 000	18 330	1670	510
Drug poisonings	4460	4334	3483	850	126
Suicide	6038	5739	5039	701	298
Inequality-related	147 346	144 164	127 013	17 150	3182
**Age-standardised mortality per 100 000**					
COVID-19 unmitigated	903	903	903	899	910
COVID-19 fully mitigated	35	35	35	35	36
Drug poisonings	7	7	6	16	7
Suicide	9	9	9	13	16
Inequality-related	253	254	245	346	210
**Impact on life expectancy (years)**					
COVID-19 unmitigated	−5.96	−5.96	−6.03	−5.26	−5.76
COVID-19 fully mitigated	−0.33	−0.33	−0.34	−0.29	−0.32
Drug poisonings	−0.20	−0.20	−0.17	−0.45	−0.22
Suicide	−0.25	−0.25	−0.24	−0.34	−0.49
Inequality-related	−3.51	−3.53	−3.40	−4.73	−3.02

GB, Great Britain; UK, United Kingdom.

The lower age distribution of suicide, drug poisoning and inequality-related deaths reduces further the difference between them and unmitigated COVID-19 when using the age-standardised and life expectancy contribution measures, such that the negative contribution to life expectancy of unmitigated COVID-19 represents the equivalent of the inequality-related deaths occurring over only 1.7 years for the UK overall. This means that over 10 years there is a greater negative life expectancy contribution from inequality than about six completely unmitigated COVID-19 pandemics for the UK overall. The estimated life expectancy impacts of different numbers of crude COVID-19 deaths are provided in online supplemental figure S2 and table S5.

## DISCUSSION

### Main results

COVID-19 represents a large and urgent mortality risk for populations across the world. However, counting cumulative crude deaths is an unhelpful means of ascertaining the scale of this risk. Comparing standardised mortality rates and life expectancy contributions of COVID-19 and three causes of death that are strongly socially determined—suicide, drug poisoning and inequality-related deaths—reveals that the mortality from a fully unmitigated COVID-19 pandemic is modelled to be responsible for the negative life expectancy contribution that occurs due to the cumulative inequality-related deaths over the course of around 1.7 years. Putting this another way, over a 10-year period if there were around six unmitigated pandemics on the scale of the current COVID-19 pandemic, the impact on life expectancy would be less than that of inequalities.

### Strengths and limitations

There is an urgency in being able to calibrate the mortality risk due to COVID-19 in order to be able to ascertain the appropriate level of control measures required. This study therefore uses the available modelled data that have been used to justify the control measures in place at the time of writing alongside routinely available and published data on other mortality risks that have been present in the UK and around the world for some time.^12^ There are, however, a number of limitations. The Imperial model upon which our study is based, does not describe an endemic infection scenario which could transpire. We have had to model the age-specific mortality of COVID-19 by 5-year age band as this was not available in the Imperial College data. The exponential relationship between age and COVID-19 mortality alongside the open upper age bound in our data make the estimates at this upper age more uncertain. Similarly, the Imperial College modelling does not provide estimates by sex, although the emerging evidence suggests that the mortality rates are higher among men. This means that the life expectancy impacts of COVID-19 provided in this paper are likely to be overestimated because men have a systematically lower life expectancy than women and so the loss of lifespan for men will be less. It is also recognised that COVID-19 mortality rates are higher among those with co-morbidities for any given age-sex group.^13^ Our modelling does not differentiate between groups on this basis and is therefore likely to be a further source of systematic bias which overestimates the mortality impact of COVID-19. For these reasons, the impacts on mortality of COVID-19 estimated here are likely to be substantially higher than reality. Our estimate of the attributable fraction of socioeconomic inequality on life expectancy does not attempt to consider the impact of specific policies to reduce inequalities, but instead estimates the total life expectancy loss attributable to socioeconomic inequality.

### How this fits with the existing literature

Other estimates of the impact on life expectancy of COVID-19 pandemic have been made which include all excess deaths (ie, including those directly due to COVID-19 and as a result of the unintended consequences of the control measures). These are, as expected, higher than our estimates of the direct impact provided here.^14^ The COVID-19 pandemic and health inequality both clearly require a radical policy response to reduce the associated mortality. Health inequalities have long been recognised as an important policy issue^12 15^ but there has been little or no progress in reducing them over at least 40 years.^16^ For COVID-19, it is also important to ensure that the physical distancing measures (the substantive difference between an unmitigated and fully mitigated COVID-19 pandemic) do not cause more population health damage than the gain achieved through mitigation.^17^ Physical distancing is likely to have marked impacts on the economy, incomes, employment, social isolation, physical activity and education. A rapid Health Impact Assessment has identified that the potential for unintended negative health consequences is very large.^18^ Although many of the relevant mechanisms and negative impacts have been reduced to some extent in the UK and elsewhere (eg, through the policy of funding part of the wages of staff who have been furloughed), it is highly unlikely that these can be reduced to zero, particularly given the scale of the economic shock.^19^

### Implications

The policy response to public health challenges should match the mortality risk. The analysis in this paper indicates that the long-term life expectancy impact of inequalities is substantially greater than even an unmitigated COVID-19 pandemic because the problem of inequalities is ongoing. The rapid policy response to COVID-19 demonstrates what governments can and should do in the face of a massive population health challenge. Yet the mortality risk from the socially generated causes compared here, as well as many others, over only a few short years contributes many more deaths on all metrics than COVID-19. It is interesting to compare the radical government action in the face of the COVID-19 threat but the lack of effective policy interventions to reduce income, wealth and power inequalities (eg, through social security benefit values, progressive taxes, ownership of capital and so on^15 20 21^) to reduce inequality-related mortality. The post-COVID-19 pandemic period should be used to ‘build back better’ and ensure that society and the economy in the future provides the basis to reduce social inequalities in health and all avoidable causes of death.^6^ Future monitoring and reporting of COVID-19 mortality should include age-standardised mortality rates and life expectancy contributions for set time periods rather than simply reporting cumulative crude deaths. The estimation of the life expectancy contribution of COVID-19 should also be repeated later in the pandemic when actual (rather than modelled) mortality data are available for the population overall and stratified by sex.

## CONCLUSION

The mortality risk from COVID-19 is substantial and if unmitigated could lead to a decline of 5.96 years of life expectancy. The risks from recurrent deaths, such as those due to inequality, will quickly surpass those due to COVID-19. Building the economy back better such that inequality-related deaths are reduced in the future is as important as physical distancing is now for future population health.

**What is already known on this subject**

- COVID-19 has been modelled to create a substantial excess mortality in the UK, depending on the degree to which this is mitigated by physical distancing measures. Best estimates of 510 000 and 20 000 crude deaths are predicted in unmitigated and fully mitigated scenarios, respectively.

**What does this study add**

- We scale the mortality impact of the modelled COVID-19 on age-standardised mortality and life expectancy against suicide, drug poisonings and inequalities. The impact of COVID-19 on life expectancy is substantial (−5.96 years) if unmitigated, but over a decade the life expectancy impact of inequalities is around six times greater than even an unmitigated pandemic.

## References

[1] *WHO Director-General’s opening remarks at the media briefing on COVID-19*. Geneva: WHO, Available https://www.who.int/dg/speeches/detail/who-director-general-s-opening-remarks-at-the-media-briefing-on-COVID-19—11-March-2020

[2] Coggon D , Rose G , Barker DJP . What is epidemiology? *Epidemiology for the uninitiated*. 4th Edition. ed. London: BMJ Publishing Group, 1997.

[3] *Global surveillance for COVID-19 caused by human infection with COVID-19 virus*. Geneva: WHO, 2020.

[4] McCartney G , Fenton L , Minton J , et al. Is austerity responsible for the recent change in mortality trends across high income nations? A protocol for an observational study. *BMJ Open* 2020;10:e034832. 10.1136/bmjopen-2019-034832 PMC7044814 31980513

[5] Geronimus AT , Bound J , Waidmann TA , et al. Drugs, and Whack-a-Mole: fundamental and proximate causes of widening educational inequity in US life expectancy by sex and race, 1990–2015. *J Health Soc Behav* 2019;60:222–39. 10.1177/0022146519849932 31190569 PMC6684959

[6] McCartney G , Hearty W , Arnot J , et al. Impact of political economy on population health: a systematic review of reviews. *Am J Public Health* 2019;109:e1–e12. 10.2105/AJPH.2019.305001 PMC6507992 31067117

[7] Ferguson NM , Laydon D , Nedjati-Gilani G , et al. *Impact of non-pharmaceutical interventions (NPIs) to reduce COVID-19 mortality and healthcare demand*. London: Imperial College, 2020. Available https://www.imperial.ac.uk/media/imperial-college/medicine/sph/ide/gida-fellowships/Imperial-College-COVID-19-NPI-modelling-16-03-2020.pdf 10.1007/s11538-020-00726-x PMC7140590 32270376

[8] *Analysis of deaths involving COVID-19: deaths occurring between March and June 2020 England and Wales*. London: ONS, 2020: Available https://www.ons.gov.uk/peoplepopulationandcommunity/birthsdeathsandmarriages/deaths/bulletins/deathsinvolvingCOVID-19bylocalareasanddeprivation/deathsoccurringbetween1marchand30june2020

[9] Walsh D , McCartney G , Minton J , et al. Mortality trends in the UK: average stalling of improvement masks worsening rates within countries and cities - a population based trend analysis BMJ Open. Submitted for Publication. In press 10.1136/bmjopen-2020-038135 PMC7646340 33154048

[10] Lewer D , Jayatunga W , Aldridge RW , et al. Premature mortality attributable to socioeconomic inequality in England between 2003 and 2018: an observational study. *Lancet Public Health* 2019;5:33–41. 10.1016/S2468-2667(19)30219-1 PMC7098478 31813773

[11] Auger N , Feuillet P , Martel S , et al. Mortality inequality in populations with equal life expectancy: Arriaga’s decomposition method in SAS, Stata, and Excel. *Ann Epidemiol* 2014;24:575–80. 10.1016/j.annepidem.2014.05.006 24970490

[12] CSDH. *Closing the gap in a generation: health equity through action on the social determinants of health. Final report of the commission on social determinants of health (CSDH)*. Geneva: WHO, 2008. 10.1016/S0140-6736(08)61690-6 18994664

[13] Banerjee A , Pasea L , Harris S , et al. Estimating excess 1-year mortality from COVID-19 according to underlying conditions and age in England: a rapid analysis using NHS heath records in 3.8 million adults. *medRxiv* 2020;3:22.20040287. 10.1101/2020.03.22.20040287

[14] Aburto JM , Kashyap R , Scholey J , et al. Estimating the burden of COVID-19 on mortality, life expectancy and lifespan inequality in England and Wales: a population-level study. *medRxiv* 2020;2020. 10.1101/2020.07.16.20155077 PMC7818788 33468602

[15] Beeston C , McCartney G , Ford J , et al. *Health inequalities policy review for the Scottish ministerial task force on health inequalities*. Edinburgh: NHS Health Scotland, 2013.

[16] Schofield L , Walsh D , Munoz-Arroyo R , et al. Dying younger in Scotland: trends in mortality and deprivation relative to England and Wales, 1981–2011. *Health Place* 2016;40:106–15. 10.1016/j.healthplace.2016.05.007 27235691

[17] McKee M , Stuckler D . If the world fails to protect the economy, COVID-19 will damage health not just now but also in the future. *Nat Med* 2020;26:640–2. 10.1038/s41591-020-0863-y 32273610

[18] Douglas M , Katikireddi SV , Taulbut M , et al. Mitigating the wider health effects of COVID-19 pandemic response. *BMJ* 2020;369:m1557. 10.1136/bmj.m1557 PMC7184317 32341002

[19] *The OBR’s coronavirus analysis*. London: Office for Budget Responsibility, 2020. Available https://cdn.obr.uk/The_OBRs_coronavirus_analysis.pdf

[20] McCartney G , Collins C , Mackenzie M . What (or who) causes health inequalities: theories, evidence and implications? *Health Policy (New York)* 2013;113:221–7. 10.1016/j.healthpol.2013.05.021 23810172

[21] Walsh D , McCartney G , Collins C , et al. History, politics and vulnerability: explaining excess mortality in Scotland and Glasgow Public Health 2017;151:1–12. 151 10.1016/j.puhe.2017.05.016 28697372

