# Supplementary material for: Scaling COVID-19 against inequalities: should the policy response consistently match the mortality challenge?
Source: J Epidemiol Community Health. 2020 Nov 2;75(4):315–20. doi: 10.1136/jech-2020-214373 (PMC7958082; doi:10.1136/jech-2020-214373)
Supplement: Supplementary data [file jech-2020-214373supp001.pdf]

Figure S1 – Regression of log(mortality) on age, based on infection fatality rates taken from Ferguson et al, used to estimate mortality rates in 5-year age bands

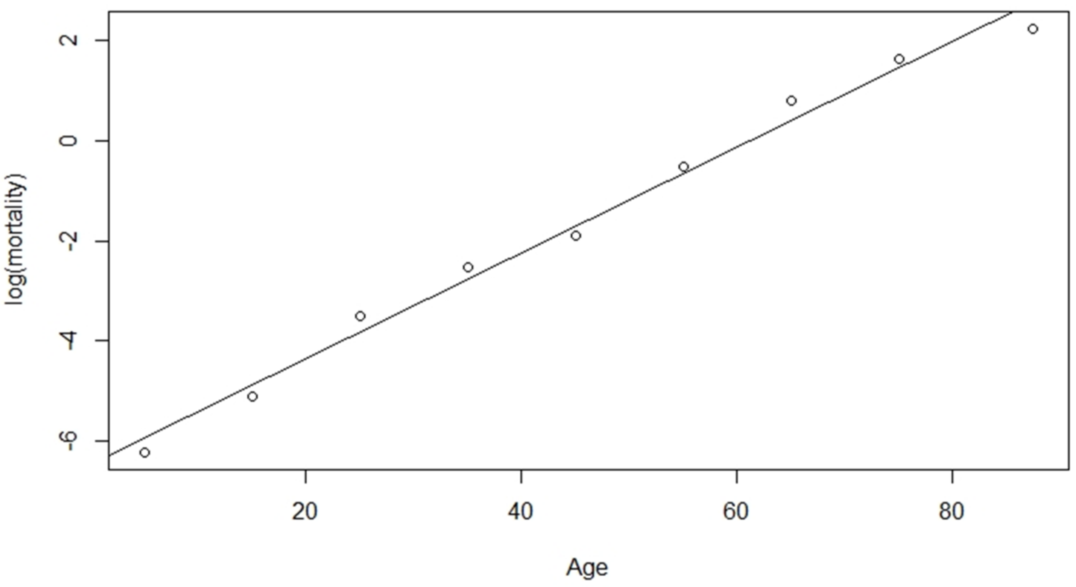

Table S1 – Estimated number of deaths due to suicide, drug- and inequality-related deaths by age (2013-2017 annualised mean, males)

Age group (years)	0-4	5-9	10-14	15-19	20- 24	25-29	30-34	35-39	40-44	45-49	50-54	55-59	60-64	65-69	70-74	75-79	80-84	85- 89	90+	Total
**Drug-related deaths**																				
**UK**	0	0	1	40	145	269	394	490	530	436	300	169	106	60	35	25	19	13	9	3,041
**GB**	0	0	1	38	134	253	379	479	519	428	293	166	105	59	34	25	18	13	9	2,956
**England & Wales**	0	0	1	29	111	200	296	383	407	341	234	141	90	51	30	22	18	13	8	2,376
**Scotland**	0	0	0	9	23	53	83	95	113	87	60	25	15	9	4	3	1	1	1	580
**Northern Ireland**	0	0	0	2	10	15	15	12	10	8	6	3	1	0	1	0	0	0	0	86
**Suicide**																				
**UK**	2	1	12	145	304	368	404	408	522	568	506	400	269	208	150	114	91	66	34	4,572
**GB**	2	1	10	134	281	341	375	384	499	543	484	385	259	201	145	111	91	65	34	4,344
**England & Wales**	2	1	9	118	249	294	328	336	434	475	427	341	231	180	129	99	85	62	31	3,832
**Scotland**	0	0	1	16	32	46	47	48	64	68	57	44	28	21	16	12	6	3	3	513
**Northern Ireland**	0	0	1	11	23	27	29	24	23	25	22	15	11	7	5	3	1	1	0	227
**Inequality**																				
**UK**	643	61	40	183	213	440	785	1,339	2,097	3,186	4,043	5,367	6,939	9,566	10,355	11,359	11,043	7,378	3,842	78,879
**GB**	628	58	38	166	179	426	764	1,280	2,059	3,085	3,920	5,238	6,749	9,318	10,078	11,155	10,875	7,228	3,755	76,998
**England & Wales**	590	56	37	137	127	345	608	1,065	1,753	2,680	3,364	4,511	5,891	8,111	8,726	9,775	9,659	6,548	3,587	67,569
**Scotland**	38	2	1	29	52	81	156	215	305	405	556	727	858	1,207	1,352	1,380	1,216	680	168	9,429
**Northern Ireland**	15	3	2	17	34	13	21	60	38	101	123	129	190	248	277	205	169	151	87	1,881

Table S2 – Estimated number of deaths due to suicide, drug- and inequality-related deaths by age (2013-2017 annualised mean, females)

Age group (years)	0-4	5-9	10-14	15-19	20- 24	25-29	30-34	35-39	40-44	45-49	50-54	55-59	60-64	65-69	70-74	75-79	80-84	85- 89	90+	Total
**Drug-related deaths**																				
**UK**	1	0	2	23	50	87	134	182	204	195	167	115	82	57	38	30	22	16	13	1,419
**GB**	1	0	2	21	47	85	130	177	200	190	163	112	79	55	38	30	22	16	13	1,378
**England & Wales**	1	0	1	17	39	71	99	133	151	149	131	91	68	49	33	27	21	14	12	1,108
**Scotland**	0	0	1	4	8	14	31	44	48	41	31	21	11	6	4	3	1	2	0	270
**Northern Ireland**	0	0	0	2	3	2	4	5	5	6	4	3	3	1	1	0	0	0	0	41
**Suicide**																				
**UK**	2	0	8	51	91	93	115	123	142	169	178	124	96	80	60	48	37	29	22	1,466
**GB**	2	0	8	48	82	87	108	116	134	160	169	119	92	77	59	47	36	29	22	1,395
**England & Wales**	1	0	6	43	72	76	94	96	112	136	147	102	78	69	52	42	33	27	21	1,207
**Scotland**	0	0	2	5	10	11	14	20	22	24	23	18	13	8	7	5	3	2	1	188
**Northern Ireland**	0	0	0	3	8	6	7	7	8	9	9	4	4	3	1	1	0	0	0	71
**Inequality**																				
**UK**	398	36	18	98	67	155	360	606	1,081	1,835	2,417	3,009	4,515	6,452	8,141	10,117	11,699	10,351	8,483	69,837
**GB**	416	35	16	90	55	147	344	588	1,051	1,758	2,358	2,910	4,430	6,275	7,993	9,881	11,536	10,231	8,413	68,526
**England & Wales**	394	36	8	87	38	103	267	479	888	1,535	2,039	2,480	3,795	5,462	6,921	8,659	10,234	9,190	8,084	60,699
**Scotland**	22	-1	8	3	17	44	77	109	162	223	319	430	635	813	1,072	1,222	1,302	1,041	329	7,827
**Northern Ireland**	-18	1	2	8	12	9	17	18	31	77	59	98	85	177	148	235	163	120	71	1,312

Table S3 – Estimated number of deaths due to inequality-related deaths by age (2013-2017 annualised mean, total population), using excess deaths over England least deprived decile for all analyses

**Age group (years)**	**0-4**	**5-9**	**10-14**	**15-19**	**20- 24**	**25-29**	**30-34**	**35-39**	**40-44**	**45-49**	**50-54**	**55-59**	**60-64**	**65-69**	**70-74**	**75-79**	**80-84**	**85- 89**	**90+**	**Total**
**Inequality**																				
UK	1,077	102	62	268	202	567	1,150	1,932	3,243	5,085	6,606	8,509	11,655	16,479	19,050	22,342	23,359	18,380	13,121	153,190
GB	1,030	97	59	252	178	544	1,114	1,886	3,169	4,964	6,441	8,302	11,379	16,084	18,588	21,781	22,804	17,926	12,760	149,358
England & Wales	986	92	51	213	143	435	910	1,576	2,669	4,248	5,512	7,090	9,838	13,797	15,830	18,561	19,657	15,579	11,373	128,558
Scotland	43	5	9	39	35	109	205	311	500	716	929	1,213	1,541	2,287	2,757	3,220	3,147	2,347	1,387	20,800
Northern Ireland	48	5	2	16	24	24	35	46	74	121	165	207	275	395	463	562	556	454	362	3,832

Table S4 – Life expectancy impacts of COVID-19 based on the age distribution of underlying COVID-19 deaths in England & Wales, March – June 2020

	UK	GB	England & Wales	Scotland	Northern Ireland
Impact on life expectancy (years)					
COVID-19 20,000 GB deaths	-0.32	-0.32	-0.32	-0.27	-0.30
COVID-19 510,000 GB deaths	-5.50	-5.50	-5.57	-4.82	-5.31

Figure S2 – Impact on life expectancy (in years) of a varying number of COVID-19 deaths in GB.

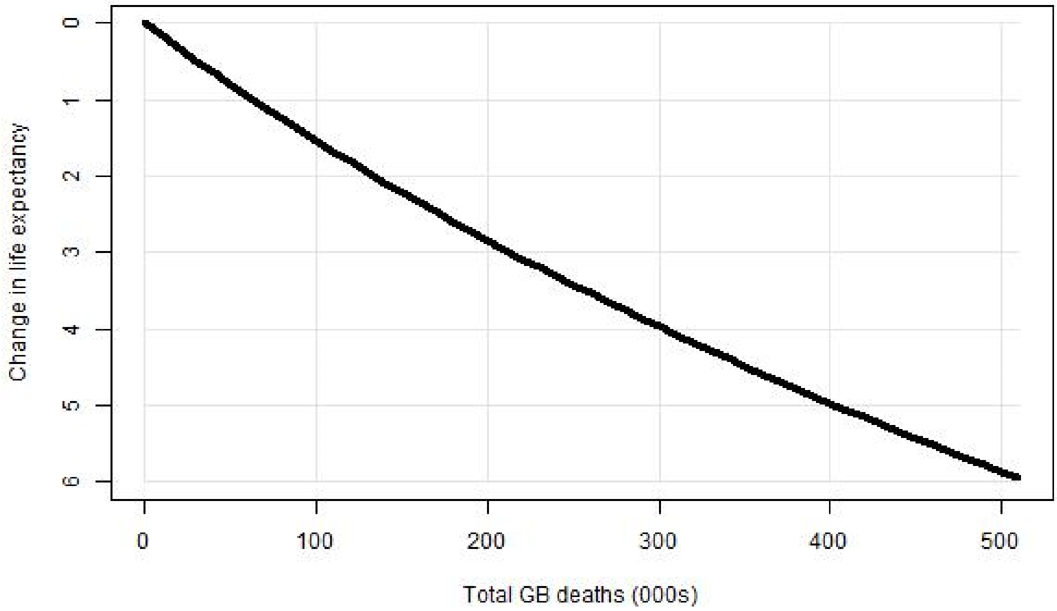

Table S5 – Estimated life expectancy impacts of 50,000 and 250,000 crude COVID-19 deaths, taken from Figure S5

	UK	GB	England & Wales	Scotland	Northern Ireland
Impact on life expectancy (years)					
COVID-19 50,000 GB deaths	-0.81	-0.81	-0.82	-0.70	-0.77
COVID-19 250,000 GB deaths	-3.42	-3.43	-3.47	-2.99	-3.30
